# *Arabidopsis Myo*-Inositol-1-Phosphate Synthases Moonlight in Nuclear Gene Regulation

**DOI:** 10.3390/plants15101454

**Published:** 2026-05-10

**Authors:** Yu Luo, Wei-Cai Yang

**Affiliations:** State Key Laboratory of Molecular and Developmental Biology, Institute of Genetics and Developmental Biology, Chinese Academy of Sciences, Beijing 100101, China

**Keywords:** *myo*-inositol-1-phosphate synthase (MIPS), moonlighting proteins, nuclear gene regulation, inositol biosynthesis, transcriptomics

## Abstract

*Myo*-inositol-1-phosphate synthase (MIPS) catalyzes the first committed step of de novo inositol biosynthesis, yet genetic evidence suggests that *Arabidopsis* MIPS proteins also have catalysis-independent functions. Although moonlighting proteins are increasingly recognized, their identification and functional dissection in plants remain limited. We asked whether the catalytic outputs of MIPS can be uncoupled from its inositol-independent functions. Here, using an inositol-rescue transcriptomic strategy, we separated catalytic inositol-biosynthetic outputs from inositol-independent functions of MIPS in *Arabidopsis* seedlings. Exogenous inositol had little effect on the wild type but extensively reprogrammed the *mips1 mips3* transcriptome without fully restoring it to the wild type state. The inositol-independent branch was associated mainly with nuclear gene-regulatory processes, with broader implications for development and immunity. By contrast, the catalytic branch was linked primarily to cellular metabolism and structural organization, with broader roles in stress responses and polar growth. These findings support a dual-function model in which *Arabidopsis* MIPS proteins couple cytosolic inositol biosynthesis with candidate moonlighting functions associated with nuclear gene-regulatory modules. More broadly, this work provides a framework for understanding how metabolic enzymes coordinate development and stress responses, and opens new avenues for exploring how plant gene duplication may foster functional innovation and adaptation to environmental change.

## 1. Introduction

Proteins with multiple biochemical activities add cellular complexity and confer adaptive advantages. Moonlighting proteins are single polypeptides that perform more than one unrelated function [1,2]. They are most often enzymes, but also include receptors, transmembrane channels, and chaperones [3]. Moonlighting can arise from changes in subcellular localization or expression, interaction with different protein or small-molecule partners, or the presence of multiple catalytic sites. To date, more than 500 moonlighting proteins have been identified, however, only a small fraction has been characterized in plants [4]. Discovering additional plant moonlighting proteins and dissecting their dual functions will reveal unexpected regulatory roles of well-studied enzymes and uncover further layers of metabolic and regulatory integration.

Intracellular inositol pool is derived from either environmental uptake or de novo synthesis by *myo*-inositol-1-phosphate synthase (MIPS). The *Arabidopsis* MIPS genes participate in diverse processes including embryo development, post-embryonic development, light-dependent cell death regulation, immune response, and abiotic stress tolerance [5,6,7,8,9], raising the possibility that their functions extend beyond inositol biosynthesis. Consistent with this idea, our previous work showed that the *mips* double mutant displays short roots and impaired cytokinesis in the absence of exogenous inositol, whereas inositol supplementation only partially rescues the short-root phenotype [10], suggesting noncatalytic, inositol-independent functions of MIPS.

Here, we used an inositol-rescue transcriptomic design to disentangle the catalytic outputs of MIPS from its candidate moonlighting functions in *Arabidopsis*. We show that exogenous inositol only partially restores the *mips1 mips3* transcriptome, thereby separating the catalytic and noncatalytic contributions of MIPS. Transcriptomic analyses associate the inositol-independent branch mainly with nuclear gene-regulatory processes, whereas the catalytic branch is linked primarily to metabolism and cellular organization. Together, these findings suggest *Arabidopsis* MIPS as a metabolic enzyme with an additional candidate moonlighting function associated with nuclear gene-regulatory processes.

## 2. Results

### 2.1. Evolutionary Expansion of Plant MIPS Genes

**Figure 1:**
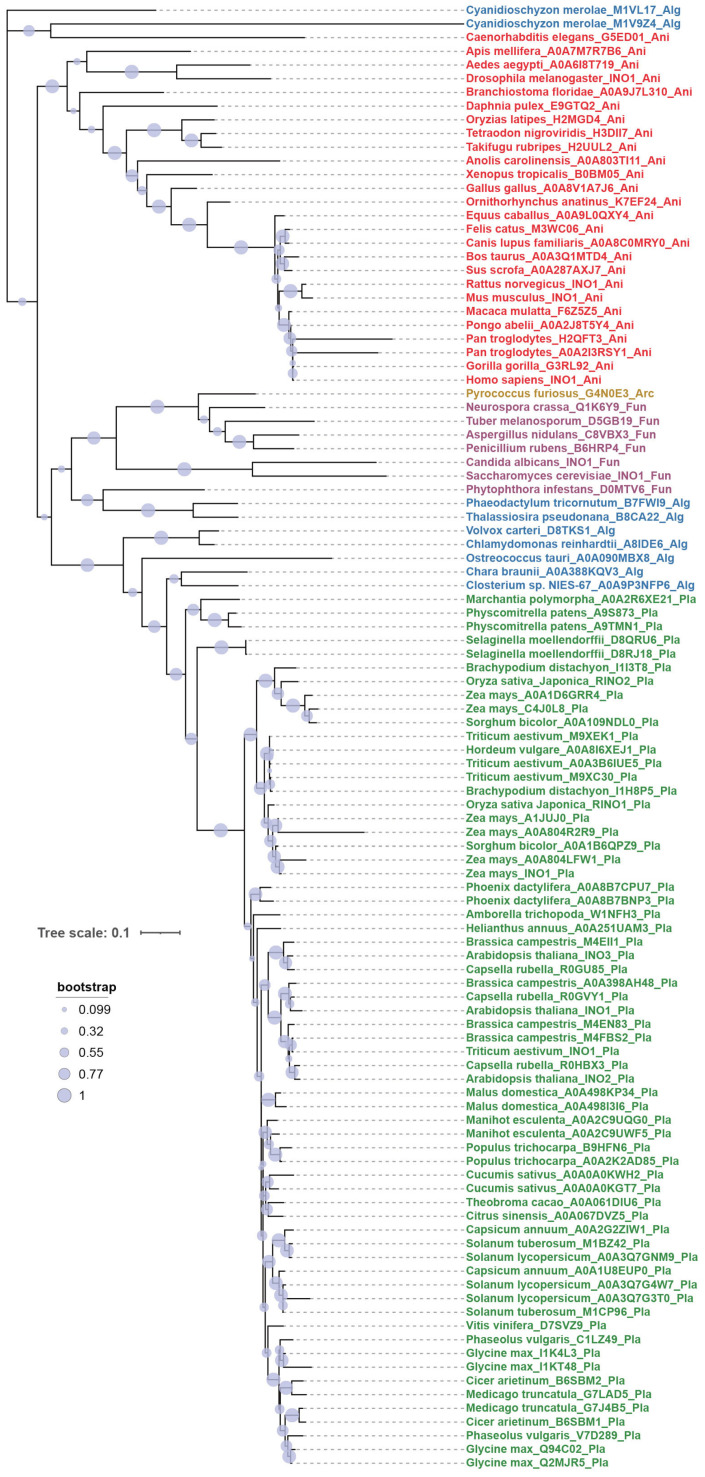
Phylogenetic relationships of MIPS proteins across plants and other taxa. A maximum-likelihood phylogeny was constructed for 107 MIPS protein sequences from 71 species. The dataset comprises 64 plant sequences (29 species), 26 animal sequences (25 species), 9 algal sequences (8 species), 7 fungal sequences (7 species) and 1 archaeal sequence (1 species). Tip labels are annotated with species name, UniProt accession and a taxonomic group code (Pla, Ani, Fun, Alg, Arc); colored labels indicate major groups (green, plants; red, animals; blue, algae; mauve, fungi; ochre, archaea). The tree was inferred under the best-fitting amino-acid substitution model with a maximum-likelihood approach and bootstrap resampling; bootstrap support values are shown at internal nodes. The scale bar indicates the number of amino-acid substitutions per site.

To assess whether MIPS proteins may have undergone functional diversification during evolution, we first examined their phylogenetic distribution and gene copy number across representative lineages. MIPS is deeply conserved across evolution, with homologs present in archaea and broadly distributed throughout eukaryotes [11]. In animals and most algae, MIPS is typically encoded by a single gene, whereas plant genomes frequently harbor multiple MIPS paralogs (Figure 1). In *Arabidopsis thaliana*, the three MIPS proteins are highly similar in sequence (>90% identity), and all three retain conserved inositol-biosynthetic activity, as previously demonstrated by functional complementation of the yeast *ino1* mutant [8]. Because exogenous inositol only partially rescues the short-root phenotype of the *mips1 mips3* mutant [10], we reasoned that MIPS may perform additional inositol-independent functions. Together, the evolutionary conservation of the catalytic core and the lineage-specific expansion of *MIPS* genes in plants provide a rationale for testing candidate moonlighting functions of *Arabidopsis* MIPS proteins. To examine this possibility more directly, we next used an inositol-rescue transcriptomic strategy to separate catalytic and inositol-independent outputs of MIPS.

### 2.2. Transcriptomic Strategy Separates Catalytic from Moonlighting Functions

**Figure 2:**
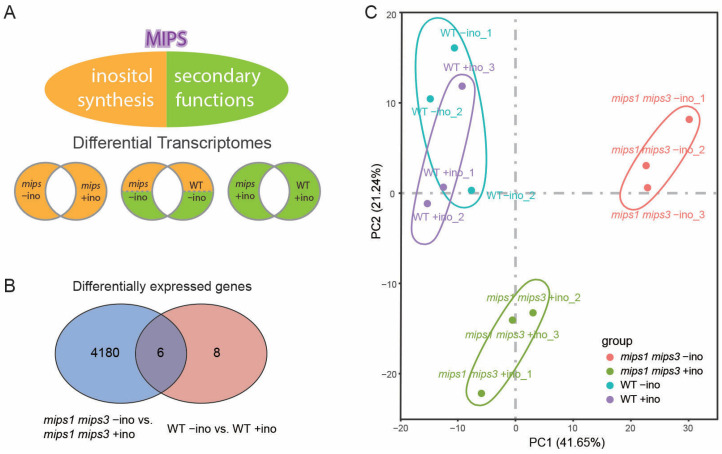
Transcriptomic strategy for separating catalytic and moonlighting functions of *Arabidopsis* MIPS. (**A**) Schematic workflow to distinguish the canonical enzymatic role of MIPS in inositol biosynthesis from its secondary (moonlighting) functions by comparing transcriptomes of WT seedlings and the *mips1 mips3* double mutant, carrying mutations in *MIPS1* and *MIPS3* genes, grown with or without inositol supplementation. (**B**) Overlap of differentially expressed genes (DEGs) among the indicated comparisons. (**C**) PCA of 12 RNA-seq libraries (four conditions, three biological replicates each), showing separation by genotype and inositol availability.

The triple mutant and the *mips1 mips2* double mutant are embryo lethal at an early stage of embryogenesis, whereas *mips1* or *mips3* single mutants show no obvious phenotype [8]. We therefore performed RNA-seq on wild type (WT) and *mips1 mips3* seedlings grown with or without exogenous inositol to distinguish catalytic from inositol-independent functions of MIPS (Figure 2A), with three biological replicates per condition, revealing high reproducibility and clear separation of samples by genotype and inositol treatment (Figures S1 and S2). This design allowed us to compare transcriptional responses to restored inositol synthesis within the mutant and, separately, to examine inositol-independent differences between WT and *mips1 mips3* under inositol supplementation. Only 14 differentially expressed genes (DEGs) were detected between WT seedlings grown with or without inositol, whereas 4186 DEGs were identified between *mips1 mips3* seedlings grown with or without inositol (Figure 2B and Figure S2D), supporting a strong genotype-dependent response to inositol. Principal component analysis (PCA) showed little separation between WT +ino and WT −ino samples, consistent with a limited response to exogenous inositol. In contrast, *mips1 mips3* samples showed clear separation between the two treatment conditions. Together, these patterns indicate that genotype is a major determinant of global transcriptomic variation, whereas the effect of inositol treatment is much more pronounced in the mutant background (Figure 2C). These results establish the transcriptomic framework used below to distinguish catalytic outputs from inositol-independent functions of MIPS.

### 2.3. Gene Expression Patterns Distinguish Catalytic and Inositol-Independent Branches of MIPS Function

**Figure 3:**
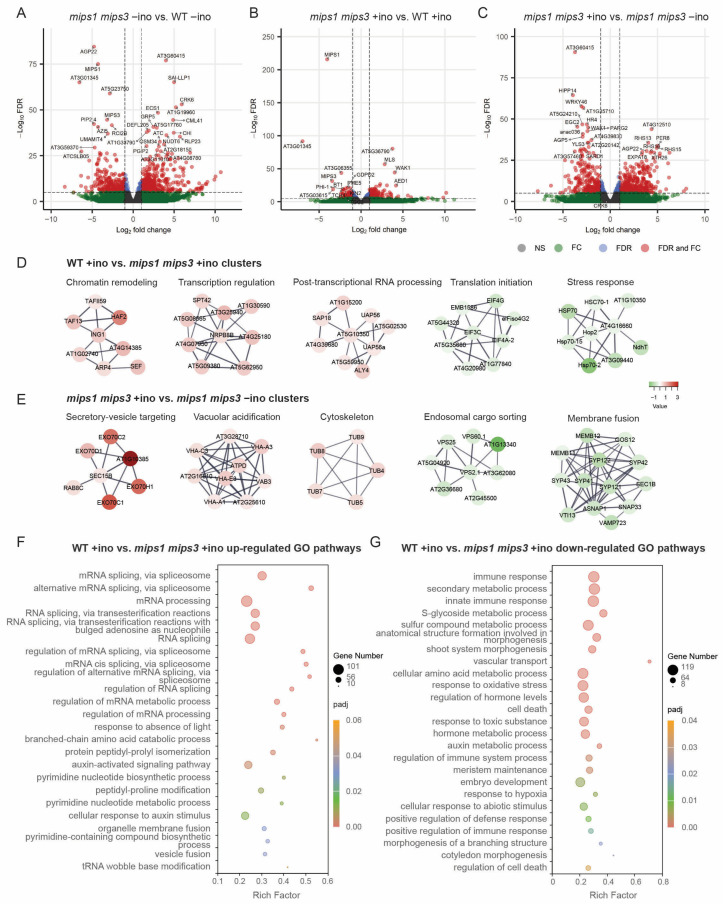
Transcriptome-based dissection of the moonlighting functions of *Arabidopsis* MIPS. (**A**–**C**) Volcano plots of DEGs for (**A**) *mips1 mips3* −ino vs. WT −ino, (**B**) *mips1 mips3* +ino vs. WT +ino, and (**C**) *mips1 mips3* +ino vs. *mips1 mips3* −ino. Dashed lines denote |log_2_fold change (FC)| = 1 (vertical) and false discovery rate (FDR; adjusted *p* value) = 0.05 (horizontal). Red: significant with |log_2_FC| ≥ 1; blue: significant with |log_2_FC| < 1; green: |log_2_FC| ≥ 1 but not significant; grey: not significant. (**D**,**E**) Protein–protein interaction subnetworks built from clustered DEGs for (**D**) WT +ino vs. *mips1 mips3* +ino and (**E**) *mips1 mips3* +ino vs. *mips1 mips3* −ino, with functional modules annotated. Node color indicates relative fold change in the corresponding comparison (*t*-test, *p* < 0.05). (**F**,**G**) Gene Ontology (GO) enrichment analysis of pathways upregulated (**F**) or downregulated (**G**) in WT +ino compared with *mips1 mips3* +ino. Rich factor is plotted on the *x*-axis, with bubble size proportional to the number of genes and color representing the adjusted *p* value (padj).

In the absence of inositol, *mips1 mips3* displayed thousands of transcriptional changes relative to WT (Figure 3A, Table S1), consistent with the diverse developmental and physiological phenotypes reported for *mips* mutants when MIPS activity is compromised [7,8]. Upon inositol supplementation, the extent of differential expression between *mips1 mips3* and WT was reduced but remained substantial (Figure 3B, Table S1), indicating that part of the mutant transcriptomic state cannot be restored by metabolic rescue alone. By contrast, the pronounced divergence between *mips1 mips3* +ino and *mips1 mips3* −ino (Figure 3C, Table S1) shows that inositol readdition triggers extensive transcriptional reprogramming in the mutant. Together, these expression patterns support the separation of a catalytic, inositol-responsive branch from an inositol-independent branch of MIPS function.

### 2.4. Network Analysis Highlights Distinct Molecular Modules Underlying the Two MIPS Branches

To gain insight into the functional basis of these expression changes, we analyzed the protein–protein interaction (PPI) network. In the WT +ino versus *mips1 mips3* +ino comparison, genes associated with the inositol-independent branch formed subnetworks related to chromatin remodeling, transcriptional regulation, and post-transcriptional RNA processing (Figure 3D). These modules were largely downregulated in the mutant, indicating coordinated repression of nuclear gene-regulatory programs in *mips1 mips3* even when inositol is supplied. Conversely, clusters related to translation initiation and stress-response proteins were upregulated in the mutant (Figure 3D).

In the *mips1 mips3* +ino versus *mips1 mips3* −ino comparison, subnetworks associated with the catalytic branch included secretory vesicle targeting, vacuolar acidification, cytoskeletal organization, endosomal cargo sorting, and membrane fusion (Figure 3E), indicating that restored inositol synthesis strongly remodels endomembrane trafficking and cytoskeletal dynamics. Thus, network analysis first resolves the molecular modules underlying the two branches, with the inositol-independent branch centered on nuclear gene-regulatory processes and the catalytic branch associated with trafficking, membrane dynamics, and cellular organization.

### 2.5. Functional Enrichment Reveals Distinct Biological Programs Associated with the Two MIPS Branches

Consistent with the network-level separation of these two branches, we next performed GO and KEGG enrichment analyses to define their broader biological programs. Under combined loss of catalytic and inositol-independent MIPS functions, genes upregulated in WT relative to *mips1 mips3* −ino were enriched for photosynthesis, carbon metabolism, lipid metabolism, cytoskeleton organization, root morphogenesis, and auxin-related pathways (Figures S3A and S4A). In contrast, genes elevated in *mips1 mips3* −ino were enriched mainly for stress- and defense-related pathways (Figures S3B and S4B).

Genes associated with the inositol-independent branch, defined by the WT +ino versus *mips1 mips3* +ino comparison, were enriched primarily for RNA processing, gene regulation, ribosome, spliceosome, and vesicular trafficking functions (Figure 3F and Figure S4C). By contrast, pathways negatively associated with this branch were enriched for stress responses, defense, secondary metabolism, and developmental programs (Figure 3G and Figure S4D).

The catalytic branch, defined by the *mips1 mips3* +ino versus *mips1 mips3* −ino comparison, was associated with induced gene programs related to cell wall remodeling, root development, trichoblast and root hair differentiation, and responses to light and gravity (Figure S3C), and broad metabolic reprogramming, including fatty acid metabolism, nucleotide sugar metabolism, glycolysis, the tricarboxylic acid cycle, and cuticular lipid biosynthesis (Figure S4E). Conversely, pathways related to immune and stress responses, hormone responses, senescence, and cell death were reduced upon inositol readdition (Figure S3D), together with spliceosome-related functions and plant–pathogen interaction pathways (Figure S4F). Together, these enrichment analyses extend the network results by showing that the inositol-independent branch is linked mainly to nuclear regulatory programs, whereas the catalytic branch is associated primarily with metabolism, membrane-related processes, and growth-associated functions.

## 3. Discussion

A broader evolutionary perspective may help explain why multifunctional enzymes such as MIPS are particularly likely to emerge in plants. Unlike animals, plant lineages have repeatedly undergone whole-genome duplication, and plant genomes often retain duplicated genes rather than eliminating them rapidly. This persistent genetic redundancy provides a substrate for innovation: paralogs can preserve core enzymatic capacity while progressively partitioning regulatory roles across organs, developmental stages, and environmental conditions. At the same time, plant life imposes selective pressures that differ fundamentally from those acting on animals. Because plants cannot buffer environmental change through behavior or movement, they must continuously adjust growth in place. This dependence on cell-wall construction, membrane trafficking, turgor-driven expansion, and stress-responsive carbon allocation may make plant duplicate genes particularly prone to acquiring context-dependent or additional regulatory functions. In this sense, moonlighting may be especially favored in plants, yet it remains far less explored than in animals. Identifying new plant moonlighting proteins is therefore important not only for understanding how new protein functions evolve, but also for revealing how metabolism is integrated with development and stress adaptation.

**Figure 4:**
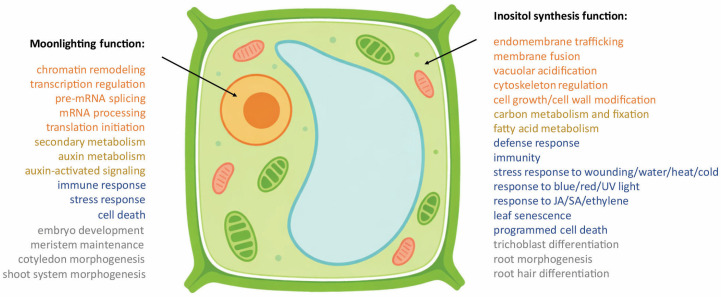
Proposed dual functional model of *Arabidopsis* MIPS proteins. Schematic summary of candidate MIPS moonlighting functions associated with nuclear gene-regulatory modules and MIPS inositol biosynthetic functions. Orange text denotes nuclear categories in the moonlighting branch and endomembrane categories in the inositol-synthesis branch, while yellow, blue, and gray text denote metabolic, stress-response, and developmental categories, respectively, in both branches.

Within this broader framework, MIPS represents an especially informative case. The inositol node occupies a central position in plant biology, contributing to membrane identity, cell-wall biogenesis, storage metabolism, and stress buffering. Multiple MIPS paralogs may therefore have been retained in plants not merely to increase enzyme dosage, but to preserve the robustness of this high-flux metabolic hub while allowing regulatory specialization in particular paralogs or cellular contexts. Our results are consistent with this view. The partial rescue of the *mips1 mips3* transcriptome by exogenous inositol indicates that the consequences of MIPS loss cannot be explained solely by impaired inositol biosynthesis, but instead reveal two separable functional branches: a canonical biosynthetic branch linked mainly to cellular organization and metabolism, and an inositol-independent branch associated with gene-regulatory processes (Figure 4). This interpretation is further supported by previous studies showing that *Arabidopsis* MIPS proteins localize to both the cytosol and nucleus [8], and that MIPS1 can participate in transcriptional regulation [9], suggesting that changes in subcellular localization may underlie the acquisition of candidate inositol-independent regulatory functions by MIPS. Together, these observations position MIPS not only as a core biosynthetic enzyme, but also as an integrative node linking metabolic state to broader regulatory control.

An important next step will be to determine how these catalytic and noncatalytic activities are coordinated across developmental stages, cell types, and environmental conditions. In particular, an important next step will be to identify nuclear interaction partners of MIPS, test whether MIPS associates with specific chromatin regions or target genes, and distinguish direct nuclear functions of MIPS from secondary transcriptional consequences of altered inositol metabolism. From an applied perspective, this dual-function view of MIPS suggests that the inositol node may be more tractable than previously appreciated: if distinct paralogs or functional branches can be selectively manipulated, it may become possible to improve stress resilience or polar growth while minimizing penalties on essential inositol metabolism and perhaps even easing growth–defense tradeoffs more precisely than by targeting downstream stress pathways alone.

## 4. Materials and Methods

### 4.1. Transcriptome Sequencing and Functional Interpretation

All *Arabidopsis thaliana* lines were in the Columbia-0 (Col-0) background. Seeds were surface-sterilized, stratified for 2 days at 4 °C, and sown on half-strength Murashige and Skoog (1/2 MS) medium (10 g L^−1^ sucrose (S0389, Sigma-Aldrich, St. Louis, MO, USA), 2.2 g L^−1^ MS basal salt mixture (M0221.0005, Duchefa Biochemie, Haarlem, The Netherlands), 0.5 g L^−1^ MES (145224-94-8, AMRESCO Chemicals, Solon, OH, USA), 9 g L^−1^ Phytagel (P8169, Sigma-Aldrich, St. Louis, MO, USA), pH 5.8) without or with 0.5 g L^−1^ *myo*-inositol (I7508, Sigma-Aldrich, St. Louis, MO, USA), as indicated. This inositol-rescue design included four groups (WT ± inositol and *mips1 mips3* ± inositol), with three biological replicates per group. Seedlings were grown vertically at 22 °C under a 16 h/8 h light–dark cycle. The *mips1 mips3* double mutant, carrying mutant alleles of *MIPS1* (AT4G39800) and *MIPS3* (AT5G10170) in the Columbia-0 background, was obtained from the previously described line reported by Luo et al. [8]. Total RNA was extracted from 7-day-old seedlings using the RNeasy Plant Mini Kit (Qiagen, Hilden, Germany). mRNA was enriched, fragmented, and reverse-transcribed, and sequencing libraries were constructed using the Fast RNA-seq Lib Prep Kit V2 (ABclonal, Wuhan, China). Libraries passing quality control were pooled and sequenced (2 × 150 bp paired-end) on an Illumina NovaSeq X Plus platform (Illumina, San Diego, CA, USA). Reads were quality-filtered with fastp [12], mapped to the *Arabidopsis* reference genome (TAIR10) using HISAT2 v2.0.5 [13], and assembled with StringTie v3.0.3 [14]. Gene-level counts were obtained with featureCounts v2.1.1 [15], and expression was summarized as FPKM.

### 4.2. Bioinformatics and Statistical Analysis

Differential expression analyses were performed with a multifactor DESeq2 v1.50.2 [16] analysis on raw counts, using the design formula ~ genotype + treatment + genotype:treatment, where genotype and inositol treatment were modeled as fixed factors. Specific contrasts among the four genotype–treatment combinations were extracted from the fitted model. GO and KEGG pathway enrichment analyses were conducted using clusterProfiler v4.0 [17]. PCA, Venn-diagram visualization, hierarchical clustering, and volcano-plot generation were performed in R to assess global transcriptional differences and visualize expression patterns among sample groups. For hierarchical clustering of transcripts, four pairwise comparisons were conducted, each involving two groups with three biological replicates per group. Transcript expression values were converted to Z-scores, and hierarchical clustering was performed using Euclidean distance and complete linkage. Volcano plots were generated using the R package EnhancedVolcano v1.0.1 [18], with thresholds of absolute log_2_FD > 1 and −log_10_FDR > 1.3 for visualization of significantly altered transcripts. Corresponding protein sequences for all expressed transcripts were obtained from the *Arabidopsis* genome annotation and used as input for protein–protein interaction analysis. Protein–protein interaction networks were inferred with the STRING v12.0 [19] and visualized and further subdivided into sub-networks using Cytoscape v3.7.1 [20].

### 4.3. Phylogenetic Tree Construction

For phylogenetic analysis, we downloaded reference proteomes from UniProt for all species included in this study and built a local BLAST database using the NCBI BLAST+ suite v2.17.0 [21]. The *Arabidopsis thaliana* MIPS1 protein was used as the query in BLASTP searches against this database, with an E-value cutoff of 1 × 10^−2^ and a minimum query coverage of 60%. For species with multiple hits, we inspected UniProt annotations v2025_04 [22] to distinguish isoforms from paralogs and retained one representative full-length sequence per gene. The resulting set of 107 MIPS homologs was aligned in MEGA X v10.0.x [23]. The best-fitting amino-acid substitution model was selected in MEGA X based on the Bayesian Information Criterion, and a maximum-likelihood tree was then inferred under the selected model with 1000 bootstrap replicates. The final tree was visualized in iTOL v5 [24], where tip labels were annotated with species name, UniProt accession and taxonomic group codes (Pla, Ani, Fun, Alg, Arc), and colored according to major taxonomic groups.

## Data Availability

All data generated or analyzed in this study are available in this article and its supplementary information. Arabidopsis genes involved in this study can be found at TAIR (www.arabidopsis.org), with the following accession numbers: MIPS1 (AT4G39800), MIPS3 (AT5G10170). All raw RNA-seq data have been deposited in the NCBI Sequence Read Archive (SRA) under BioProject accession PRJNA1423463.

## Supplementary Materials

The following supporting information can be downloaded at: https://www.mdpi.com/article/10.3390/plants15101454/s1, Supplementary Figure S1. Correlation matrix of Pearson correlation coefficients among all samples; Supplementary Figure S2. Hierarchical clustering of differentially expressed genes between paired conditions. Heatmaps show hierarchical clustering of selected differentially expressed genes (row Z-scores) between: (A) *mips1 mips3* −ino and WT −ino, (B) *mips1 mips3* +ino and WT +ino, (C) *mips1 mips3* +ino and *mips1 mips3* −ino, and (D) WT +ino and WT −ino, each with three biological replicates per condition; Supplementary Figure S3. GO enrichment analysis of DEGs in response to *MIPS* mutation and inositol supplementation. Bubble plots show enriched biological pathways for upregulated and downregulated DEGs in the indicated comparisons: (A,B) WT −ino vs. *mips1 mips3* −ino (A, upregulated; B, downregulated) and (C,D) *mips1 mips3* +ino vs. *mips1 mips3* −ino (C, upregulated; D, downregulated). The *x*-axis represents the rich factor. Bubble size indicates the number of genes, and color denotes the padj; Supplementary Figure S4. KEGG pathway enrichment of DEGs associated with inositol availability and MIPS function. Bubble plots show enriched KEGG pathways among upregulated and downregulated DEGs in the indicated comparisons: (A,B) WT −ino vs. *mips1 mips3* −ino (A, upregulated; B, downregulated); (C,D) WT +ino vs. *mips1 mips3* +ino (C, upregulated; D, downregulated); and (E,F) *mips1 mips3* +ino vs. *mips1 mips3* −ino (E, upregulated; F, downregulated). The *x*-axis shows the rich factor; bubble size reflects gene count, and color indicates the padj; Supplementary Table S1. Differentially expressed genes identified in the indicated pairwise transcriptomic comparisons; Supplementary Table S2. Summary of RNA-seq library, sequencing, and mapping metrics for all samples.
