# Supplementary material for: Arabidopsis Myo-Inositol-1-Phosphate Synthases Moonlight in Nuclear Gene Regulation
Source: Plants (Basel). 2026 May 10;15(10):1454. doi: 10.3390/plants15101454 (PMC13210883; doi:10.3390/plants15101454)
Supplement: Supplementary file 1 [file plants-15-01454-s001.zip › Supplementary Figures S1-S4.pdf]

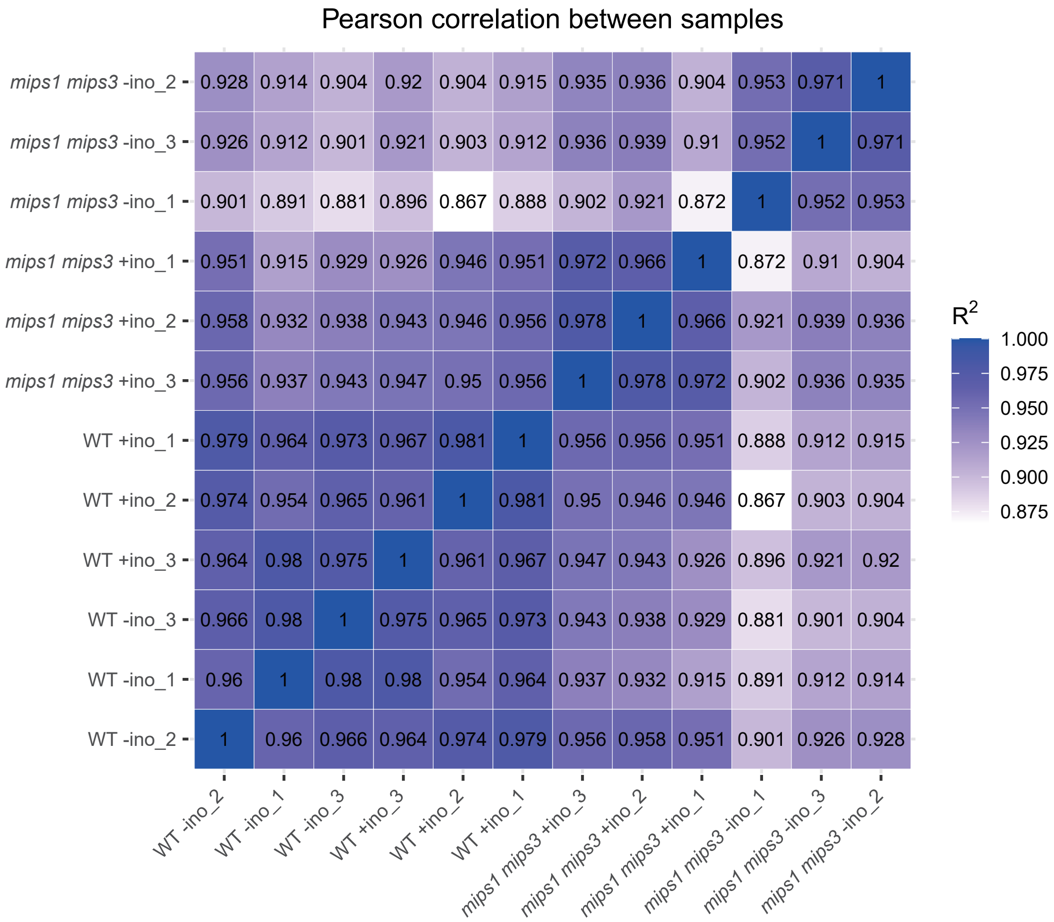

Supplementary Figure 1. Correlation matrix of Pearson correlation coefficients among all samples.

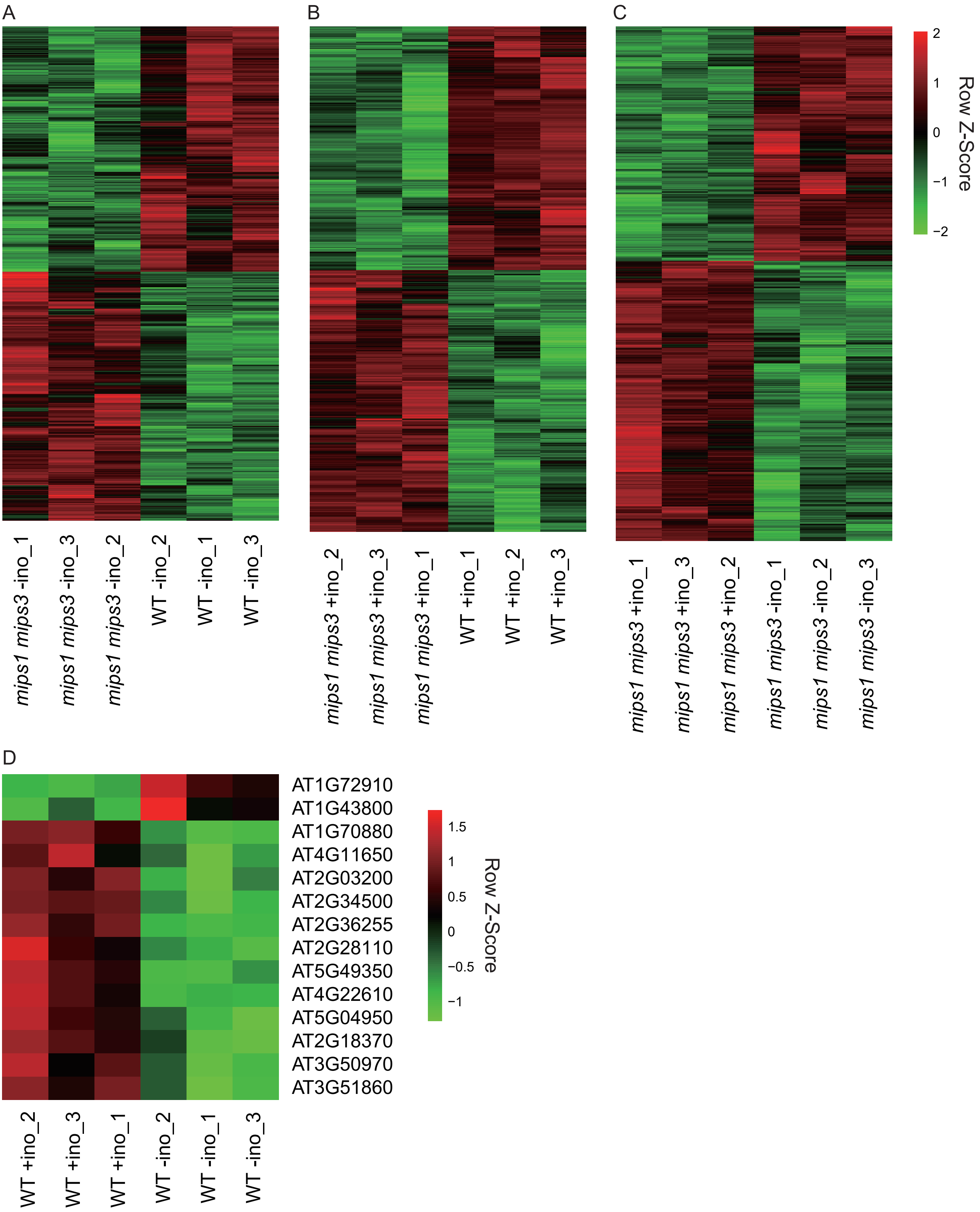

Supplementary Figure 2. Hierarchical clustering of differentially expressed genes between paired conditions. Heatmaps show hierarchical clustering of selected differentially expressed genes (row Z-scores) between: **(A)** *mips1 mips3* −ino and WT −ino, **(B)** *mips1 mips3* +ino and WT +ino, **(C)** *mips1 mips3* +ino and *mips1 mips3* −ino, and **(D)** WT +ino and WT −ino, each with three biological replicates per condition.

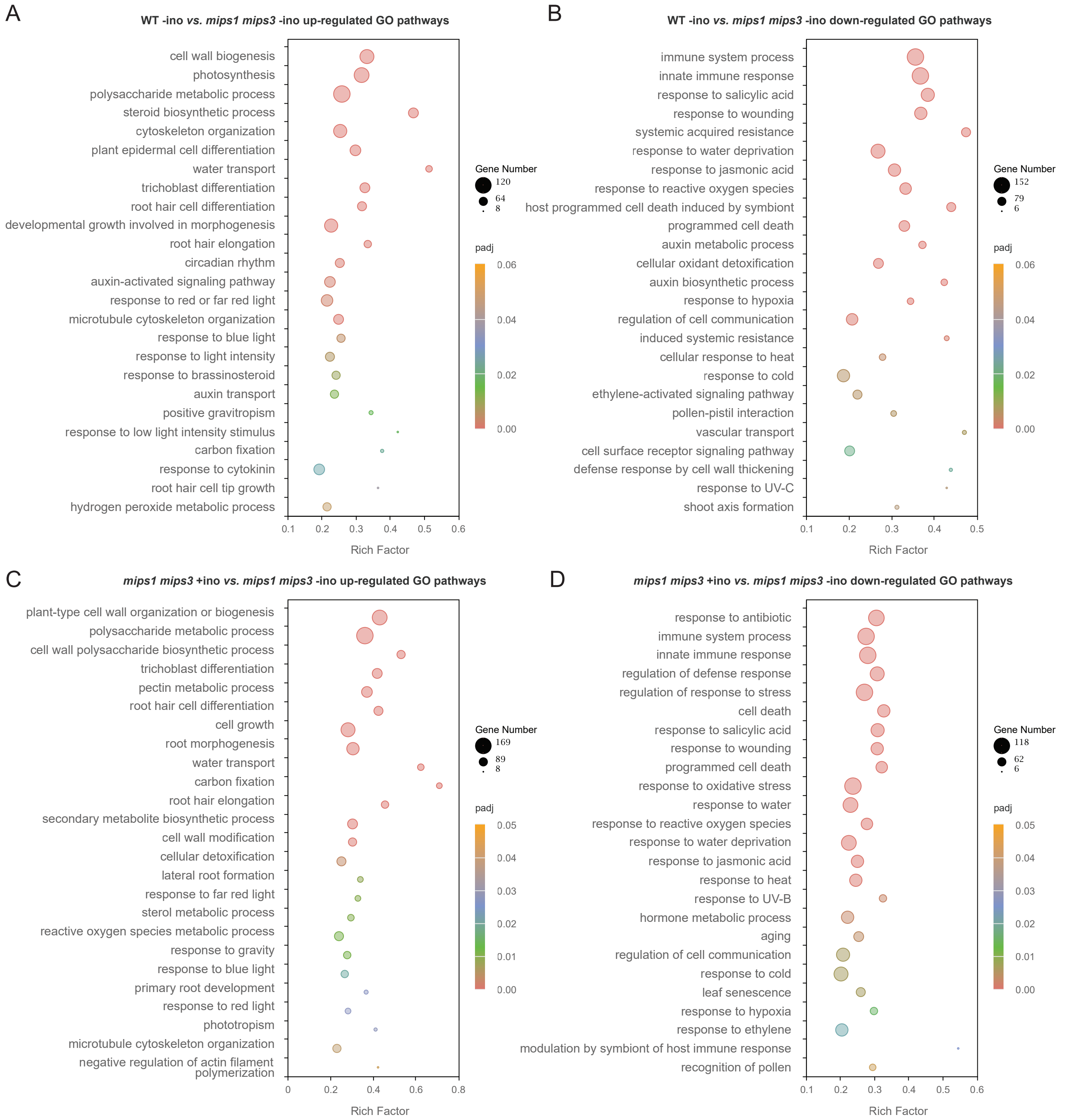

Supplementary Figure 3. GO enrichment analysis of DEGs in response to *MIPS* mutation and inositol supplementation. Bubble plots show enriched biological pathways for upregulated and downregulated DEGs in the indicated comparisons: **(A,B)** WT −ino vs. *mips1 mips3* −ino (**A**, upregulated; **B**, downregulated) and **(C,D)** *mips1 mips3* +ino vs. *mips1 mips3* −ino (**C**, upregulated; **D**, downregulated). The x-axis represents the rich factor. Bubble size indicates the number of genes, and color denotes the padj.

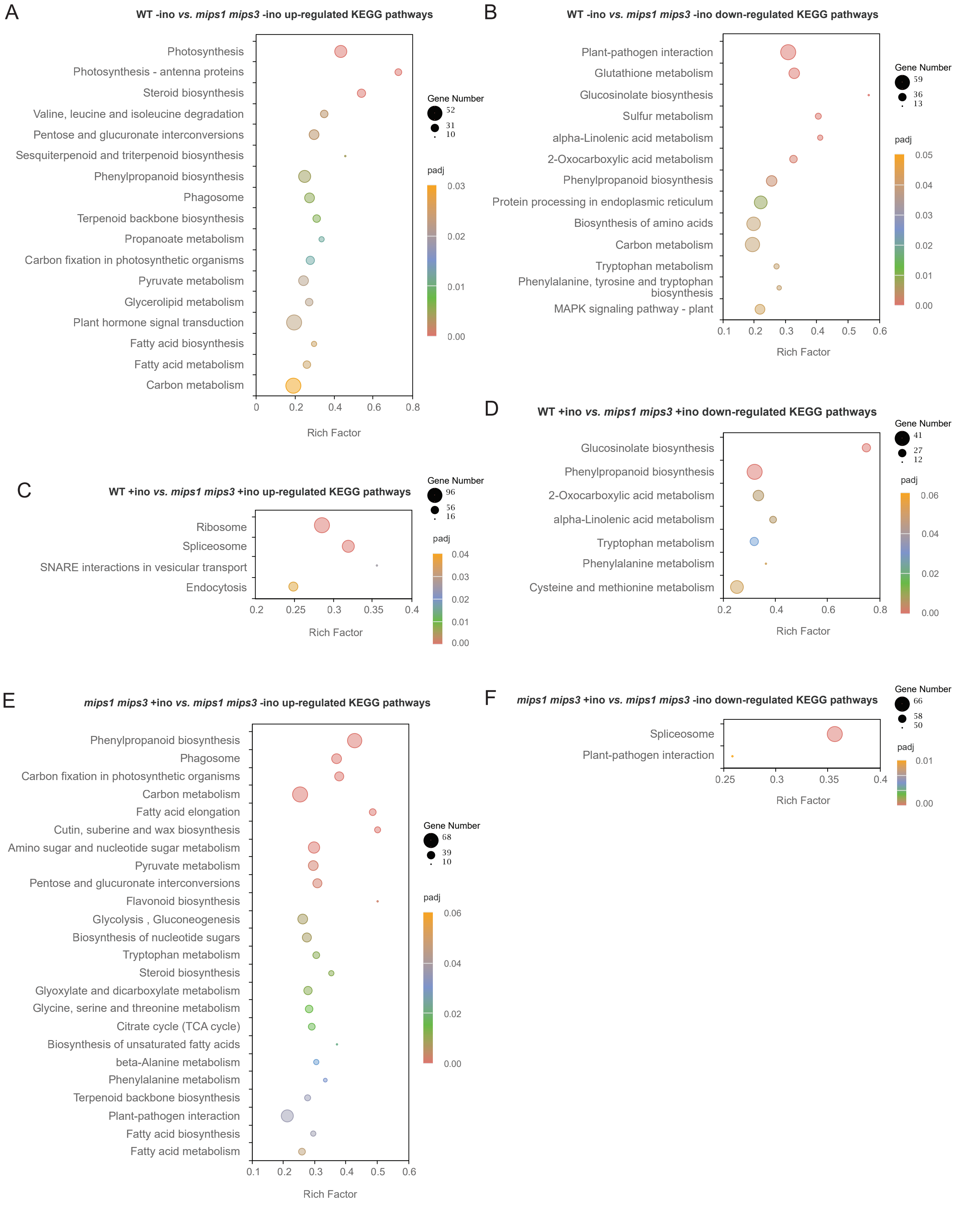

Supplementary Figure 4. KEGG pathway enrichment of DEGs associated with inositol availability and MIPS function. Bubble plots show enriched KEGG pathways among upregulated and downregulated DEGs in the indicated comparisons: **(A,B)** WT −ino vs. *mips1 mips3* −ino (**A**, upregulated; **B**, downregulated); **(C,D)** WT +ino vs. *mips1 mips3* +ino (**C**, upregulated; **D**, downregulated); and **(E,F)** *mips1 mips3* +ino vs. *mips1 mips3* −ino (E, upregulated; F, downregulated). The x-axis shows the rich factor; bubble size reflects gene count, and color indicates the padj.
